# Early Childhood Development and Schooling Attainment: Longitudinal Evidence from British, Finnish and Philippine Birth Cohorts

**DOI:** 10.1371/journal.pone.0137219

**Published:** 2015-09-09

**Authors:** Evan D. Peet, Dana C. McCoy, Goodarz Danaei, Majid Ezzati, Wafaie Fawzi, Marjo-Riitta Jarvelin, Demetris Pillas, Günther Fink

**Affiliations:** 1 Department of Global Health and Population, T. H. Chan School of Public Health, Harvard University, Boston, Massachusetts, United States of America; 2 RAND Corporation, 4570 Fifth Ave #600, Pittsburgh, Pennsylvania, United States of America; 3 Graduate School of Education, Harvard University, Cambridge, Massachusetts, United States of America; 4 University of London Imperial College of Science, Technology & Medicine, Department Epidemiology & Biostatistics, MRC PHE Centre for Environment & Health, School of Public Health, London, United Kingdom; 5 University of Oulu, Institute of Health Sciences, Oulu, Finland; 6 University of Oulu, Biocenter Oulu, Oulu, Finland; 7 Oulu University Hospital, Unit of Primary Care, Oulu, Finland; TNO, NETHERLANDS

## Abstract

**Background:**

While recent literature has highlighted the importance of early childhood development for later life outcomes, comparatively little is known regarding the relative importance of early physical and cognitive development in predicting educational attainment cross-culturally.

**Methods:**

We used prospective data from three birth cohorts: the Northern Finland Birth Cohort of 1986 (NFBC1986), the 1970 British Cohort Study (BCS1970), and the Cebu Longitudinal Health and Nutrition Survey of 1983 (CLHNS) to assess the association of height-for-age z-score (HAZ) and cognitive development measured prior to age 8 with schooling attainment. Multivariate linear regression models were used to estimate baseline and adjusted associations.

**Results:**

Both physical and cognitive development were highly predictive of adult educational attainment conditional on parental characteristics. The largest positive associations between physical development and schooling were found in the CLHNS (β = 0.53, 95%-CI: [0.32, 0.74]) with substantially smaller associations in the BCS1970 (β = 0.10, 95% CI [0.04, 0.16]) and the NFBC1986 (β = 0.06, 95% CI [-0.05, 0.16]). Strong associations between cognitive development and educational attainment were found for all three cohorts (NFBC1986: β = 0.22, 95%-CI: [0.12, 0.31], BCS1970: β = 0.58, 95%-CI: [0.52, 0.64], CLHNS: β = 1.08, 95%-CI: [0.88, 1.27]). Models jointly estimating educational associations of physical and cognitive development demonstrated weaker associations for physical development and minimal changes for cognitive development.

**Conclusion:**

The results indicate that although physical and cognitive early development are both important predictors of educational attainment, cognitive development appears to play a particularly important role. The large degree of heterogeneity in the observed effect sizes suggest that the importance of early life physical growth and cognitive development is highly dependent on socioeconomic and institutional contexts.

## Introduction

Critical to labor market outcomes and quality of life, education is the single most important predictor of individual well-being and societal development [1,2]. From an individual or societal investment perspective, the returns to education are substantial—estimates for the labor market returns to each additional year of schooling typically vary between 6 and 12% [3,4]. Education also produces positive societal benefits through improvements in peer interactions [5], reductions in crime [6], and reductions in risky behaviors including drug use [7].

While a growing literature highlights the importance of early life conditions [8,9,10], understanding the relative contributions of multiple domains of early childhood development (ECD) to educational attainment is still limited. Early life nutritional, environmental, socio-economic, and other conditions are known to impact early physical development and predict educational attainment [10,11,12,13,14], but data measuring both early cognitive development and later-life educational attainment are scarce.

Most of the existing literature linking early life experiences to later life outcomes has relied on physical growth delays (stunting) to proxy for cognitive and other domains of ECD. Physical development measured by height predicts both concurrent and future welfare [15,16] through a direct effect and through associations with other domains of development. Physical and cognitive development are correlated and reflect the interaction between biology [17], and environmental investments [15,18,19,20] or insults [19,21]. While the direct impact of physical development on educational attainment has been assessed [22,23,24], the relationship between early physical and cognitive development remains unclear and the link between early cognitive development and schooling has not been quantified.

Evidence concerning the links between physical and cognitive development and schooling is scarce because of limited prospective data containing measures of ECD and years of schooling. In this paper, we used data from three of the most comprehensive studies containing measures of ECD and schooling in order to assess the relative importance of early physical and cognitive development for educational attainment.

## Methods

### Ethics Statement

The human subject data from the three cohorts of this study was analyzed anonymously.

### Cohorts

We used data from the 1970 British Cohort Study (BCS1970), the 1983–84 Cebu Longitudinal Health and Nutrition Survey from the Philippines (CLHNS) and the 1985–86 Northern Finland Birth Cohort (NFBC1986). The BCS1970 is an ongoing longitudinal study of individuals born in England, Scotland or Wales between April 5th and April 11th, 1970 [25]. The NFBC1986 tracks mothers and their children living in Oulu and Lapland provinces who had expected dates of delivery between July 1st, 1985 and June 30th, 1986 [26]. The CLHNS randomly sampled 33 barangay, or neighborhoods, in Metro Cebu of the Philippines and women who gave birth between May 1, 1983, and April 30, 1984 composed the sample [27]. Table 1 presents additional information regarding each survey.

**Table 1:** Cohort Data Description.

Cohort Data Description														
	Location(s)	Public or private data	Baseline survey year/latest survey year	Total number of survey waves	Baseline sample size	Obs. missing height	Obs. missing cognition	Obs. missing years of schooling	Obs. missing potential confounders	Final sample size	Child age in years at early life height measure	Child age in years at early life cognition measure	Available early life cognition measures	Supplementary data
Northern Finland Birth Cohort (NFBC1986)	Oulu and Lapland provinces, Finland	Private	1986 / 2002	4	9,679	5,103	1,313	1,960	3,177	4,003	2, 5*	8	Parental report of spatial and temporal conceptual understanding	National Educational Registry from the Central Statistical Office
1970 British Cohort Study (BCS1970)	England, Scotland, Wales and North Ireland	Public	1970 / 2005	7	17,185	4,895	4,157	7,561	4,467	6,533	2, 5*	5	Surveyor administered copy designs score	
Cebu Longitudinal Health and Nutrition Survey (CLHNS)	Metropolitan Cebu, Philippines	Public	1983 / 2009	20	3,327	664	1,179	1,265	256	1,886	2	8	Surveyor administered non-verbal IQ test	

*—Height was available at both age 2 and age 5 in the NFBC1986. Height was measured on a subsample of the BCS1970 cohort at age 2 and the full sample at age 5. The main analyses used height at age 2 z-score for both the NFBC1986 and the CLHNS and used the average of height at ages 2 and 5 z-scores for the BCS1970. Alternative specifications using height at age 5 z-score and the mean of the z-scores for height at ages 2 and 5 in the NFBC1986 were also assessed and the results do not differ substantially.

### Physical development

Height, the most commonly used measure of physical development, was measured for all cohorts prior to age 5. In each cohort raw height measures were recorded in centimeters and have been converted to height-for-age z-score (HAZ) units by the most recent international reference curves produced by the World Health Organization (WHO) in 2007 and based on national cross-sectional anthropometric data from the US National Center for Health Statistics. For the NFBC1986 cohort, height was measured in 1988 and 1991 when the children were ages 2 and 5. Height was measured in 1972 on a subsample of children age 2 in the BCS1970 and in 1975 on the full sample of BCS1970 children age 5. In the CLHNS, height was measured in 1985–86 when the children were age 2. Previous evidence suggests that HAZ at age 2 is a preferable measure of early physical development because of the increasing variance in the reference group distribution by age [28,29]. Consequently, we used HAZ at age 2 when available (full samples of the NFBC1986 and CLHNS cohorts, and the 1972 subsample of the BCS1970 cohort) and supplemented with HAZ at age 5 when height is not observed at age 2 (1975 subsample of the BCS1970 cohort not observed at age 2).

### Cognitive development

Cognitive development was measured differently in each cohort with measures for the present analysis selected for maximum comparability. The measures of cognition used in the analysis of each cohort capture similar dimensions of children’s non-verbal reasoning.

The measures of cognitive development in the NFBC1986 were produced by parental assessment of child’s spatial and temporal understanding obtained via questionnaire mailed in 1993 when children were 7–8 years old. Parents were asked to report their child’s understanding of spatial and temporal concepts at below, equal, or above average levels. This measure of cognition was chosen for comparability to other the measures of cognition available in the BCS1970 and CLHNS. Additionally, factor analysis of all reported measures indicated high loadings of reported spatial and temporal understanding on a general cognitive factor, which was distinct from factors represented by other measures.

For the BCS1970, tests of cognitive abilities were performed in 1975 on 5-year-old children. The Copying Designs test asked for two copies of eight designs [30]. The Human Figure Drawing test asked for ‘‘a picture of a man or lady.” When children finished, they were asked what the drawing was, what various parts of the drawing were and to label them. Subsequently, subjects drew another picture of the opposite sex as a measure of intellectual maturity [31]. The Profile test asked children to complete a profile of a human head and face. Both the Copying Design and Human Figure Drawing test were highly correlated. Factor analysis was performed to establish the presence of a general cognitive factor among the various measures. Amongst the three tests, the Copying Designs test loaded highest on general cognitive ability, followed by the Human Figure Drawing test and the Profile test. As a result of the correlations and factor analysis, our analysis used only the Copying Designs test. Furthermore, the Copying Designs test demonstrated the most conceptual similarity to the tests or reports of cognitive abilities available in the other cohorts.

CLHNS surveyors obtained only one measure of cognitive development at or prior to the age of 8: the Philippine Non-Verbal Intelligence test. The Philippine Non-Verbal Intelligence test assessed fluid ability (i.e., analytic or reasoning skills) and was adapted specifically for the CLHNS survey [32]. The test included 100 cards, each with five drawings of culturally appropriate objects including shapes, farm animals, and familiar activities, where one of the five objects differed in a meaningful way. Children were asked to identify the different object. No time limits were given and the difficulty increased as children advanced through the test.

For each cohort, the measures of cognitive development were standardized with the sample mean equal to 0 and the standard deviation equal to 1.

### Educational Attainment

In each of the cohorts educational attainment was defined by the number of years of schooling to obtain the individual’s highest qualification. Different from the BCS1970 and CLHNS, educational qualification information in the NFBC1986 was obtained by linking a unique national identifier to the national education registry. For the 1985–86 NFBC1986 birth cohort, compulsory schooling began at age 7 and continued until age 16. The first qualification was potentially obtained at age 16 and subsequent qualifications resulted from three additional years of either vocational or upper secondary school attendance. Four years of tertiary education at either a university of applied science or a traditional university followed both vocational and upper secondary school. Post-graduate schooling and training for advanced degrees took place beginning at age 23, varying in length by degree. Given the educational system and individual information on the highest qualification, the years of completed education were derived.

For the BCS1970 cohort, qualifications were obtained through testing and completion of advanced degrees. At the time in the UK, primary school ran from ages 4 to 11, followed by secondary school until age 16. For the cohort born in 1970, education was compulsory until age 16 at which time the first qualification was potentially obtained: the Certificate of Secondary Education (CSE), or as it would later be known, the General Certificate of Secondary Education (GCSE), or the O-level. The Sixth Form level of education took place from ages 16 to 18 and prepared the children for the A-level exams. Between ages 18 and 22 the UK educational system divided into the vocational and collegiate tracks. Post-graduate schooling and training for advanced degrees took place beginning at age 23, varying in length by degree. Derivation of the years of education given the education system and available individual qualification information was determined through consultation with the Centre for Longitudinal Studies at the Institute of Education.

Children were asked their highest completed education during each wave of the CLHNS. In the 2002 and 2005 surveys the responses ranged from no school to 5 years of college. In subsequent tracking surveys, the responses included post-graduate years of education. Because of either temporary or permanent attrition, not every individual was observed in each survey between 2002 and 2009. Consequently, the years of education variable was derived from the maximum of the observed years of education between the years 2002 and 2009.

### Potential Confounders

Covariates in the analysis were chosen and included for consistency across cohorts and to represent spatial, socioeconomic, and biological influences. Spatial indicators representing regions were only included for the BCS1970 and NFBC1986. While the CLHNS sampled from one metropolitan area, the BCS1970 and the NFBC1986 contained observations from multiple regions throughout each nation. 11 indicators for the BCS1970 represented London, Scotland, York and others, while 2 indicators for the NFBC1986 represented Lapland and Oulu.

Socioeconomic measures of each household at baseline included mothers’ and fathers’ highest educational grade attained, as well as social class indicators based on fathers’ occupation. Similar to the child’s years of education, parental years of education was derived from the highest reported qualification. Social class was divided into 6 categories and derived from reported main occupation in each of the three surveys. The 6 categories are: professional/manager, non-manual skilled laborer, manual skilled laborer, semi-skilled manual laborer, unskilled manual laborer, and other (which included the unemployed).

Biological factors in physical and cognitive development included individual and family level indicators. At the individual level, an indicator of child low birth weight (weight at birth less than 2500 grams) was included. At the family level, mother’s height (in centimeters), the number of previous pregnancies the mother has had, the mother’s age at childbirth, and the mother’s smoking behavior during pregnancy (binary indicator equal to 1 if the mother smoked at all during pregnancy) were the remaining biological covariates.

### Statistical Analyses

**Fig 1:**
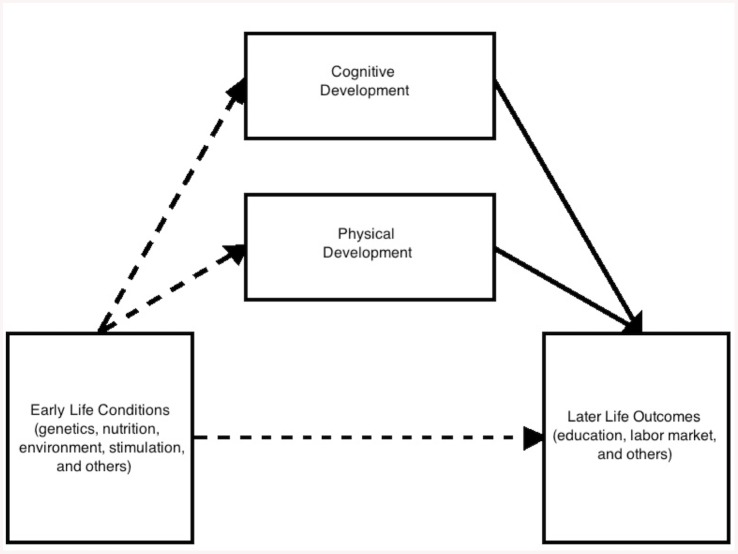
Conceptual Model. The model links early life conditions, development and later life outcomes. The analysis focuses on the bolded lines, the links between physical and cognitive development and later life educational attainment.

The conceptual model in Fig 1 displays later life outcomes including educational attainment as both directly impacted by early life conditions and indirectly affected through the impact of early life conditions on the domains of ECD. The analysis first estimated the association between physical development (HAZ) and later life completed years of schooling. Second, the association between cognitive development (standardized measure of cognition specific to each cohort) and later life completed years of schooling was estimated. Third, joint associations between physical and cognitive development and educational attainment were estimated for each of the cohorts. For each analysis and cohort, three specifications—baseline, minimal adjustment, full adjustment—were employed in order to examine the sensitivity of the estimates. The baseline specification included a gender indicator and regional fixed effects. The minimally adjusted specification added socioeconomic indicators—parental education and social class indicators. The fully adjusted specification added biological measures—child low birth weight indicator, mother’s height, mother’s number of previous pregnancies, mother’s age at childbirth, and mother’s smoking behavior during pregnancy. Consequently the estimation draws on comparisons between children of similar biological characteristics.

Furthermore, in order to assess the shape of the relationship between physical and cognitive development and years of schooling, we fitted and graphed non-parametric local polynomial models separately for each cohort. All estimates and graphs were produced using STATA version 13 (College Station, TX, USA).

## Results

In Table 2 we provide summary statistics of the dependent variable (years of education by highest grade attained), independent variables of interest (height-for-age z-score between ages 2–5 and standardized cognitive test score between ages 5–8), and each potential confounder. The distributions of HAZ among the NFBC1986 and BCS1970 cohorts were relatively similar while the distribution of HAZ in the CLHNS was skewed downward. Reported smoking during pregnancy was more prevalent among mothers in the BCS1970 and NFBC1986 cohorts than the CLHNS. The incidence of mothers and fathers obtaining more than secondary levels of education was much more common in the NFBC1986 than the BCS1970 and CLHNS. Similar to the cohort children, the distribution of mother’s height was skewed downwards in the CLHNS cohort. The number of prior pregnancies was much higher in the CLHNS than the NFBC1986 and BCS1970 and there were more mothers under 20 in the CLHNS.

**Table 2:** Summary Statistics.

Summary Statistics						
	1986 Northern Finland Birth Cohort (NFBC1986)		1970 British Cohort Study (BCS1970)		1983–84 Cebu Longitudinal Health and Nutrition Survey (CLHNS)	
	(n = 4,003)		(n = 6,533)		(n = 1,886)	
	No.	(%)	No.	(%)	No.	(%)
***Child***						
Male Gender	1947	49%	3117	48%	997	53%
Low Birth Weight (<2500 grams)	127	3%	343	5%	220	12%
Height for age: severely stunted (>3 SD below international median)	5	0%	70	1%	354	19%
Height for age: moderately stunted (>2 and < = 3 SD below international median)	39	1%	214	3%	648	34%
Height for age: mildly stunted (< = 1 SD above international median)	1649	41%	3793	58%	858	45%
Height for age: non-stunted	2238	56%	2456	38%	26	1%
Std. cognitive test: >2 SD below cohort mean	92	2%	21	0%	59	3%
Std. cognitive test: >0 and < = 2 SD below cohort mean	1265	32%	2878	44%	910	48%
Std. cognitive test: >0 and < = 2 SD above cohort mean	2576	64%	3628	56%	860	46%
Std. cognitive test:>2 SD above cohort mean	0	0%	0	0%	34	2%
Primary education or none (< = 6 years)	0	0%	0	0%	227	12%
Secondary education (>6 and < = 12 years)	2586	65%	3739	57%	873	46%
Tertiary education (>12 and < = 16 years)	1103	28%	1609	25%	605	32%
Post-graduate education (>16 years)	314	8%	1185	18%	181	10%
***Mother***						
Smoked during pregnancy	910	23%	2791	43%	265	14%
Education: primary education or none (< = 6 years)	19	0%	28	0%	1099	58%
Education: secondary education (>6 and < = 12 years)	1519	38%	5776	88%	545	29%
Education: tertiary education (>12 and < = 16 years)	1468	37%	602	9%	235	12%
Education: post-graduate education (>16 years)	997	25%	127	2%	7	0%
Height: < = 150cm	51	1%	262	4%	903	48%
Height: >150cm and < = 160cm	1284	32%	2884	44%	910	48%
Height: >160cm and < = 170cm	2328	58%	2959	45%	72	4%
Height: >170cm	340	8%	428	7%	1	0%
Previous pregnancies: none	1344	34%	2230	34%	340	18%
Previous pregnancies: 1	1330	33%	2201	34%	369	20%
Previous pregnancies: 2	740	18%	1087	17%	361	19%
Previous pregnancies: 3–4	365	9%	797	12%	453	24%
Previous pregnancies: 5–9	188	5%	207	3%	324	17%
Previous pregnancies: 10+	36	1%	11	0%	39	2%
Age at childbirth: < = 20 years old	228	6%	749	11%	342	18%
Age at childbirth: >20 and < = 30 years old	2588	65%	4509	69%	1106	59%
Age at childbirth: > = 30 and <40 years old	1116	28%	1195	18%	399	21%
Age at childbirth: >40 years old	71	2%	80	1%	39	2%
***Father***						
Education: primary education or none (< = 6 years)	0	0%	22	0%	1028	55%
Education: secondary education (>6 and < = 12 years)	1524	38%	5542	85%	577	31%
Education: tertiary education (>12 and < = 16 years)	1741	43%	648	10%	277	15%
Education: post-graduate education (>16 years)	738	18%	321	5%	4	0%
Social class/occupation: professional	551	14%	1232	19%	58	3%
Social class/occupation: non-manual skilled	848	21%	916	14%	232	12%
Social class/occupation: manual skilled	1932	48%	3114	48%	1068	57%
Social class/occupation: semi-skilled	371	9%	869	13%	240	13%
Social class/occupation: unskilled	263	7%	306	5%	189	10%
Social class/occupation: other, unemployed	38	1%	96	1%	99	5%

* Height was available for the full sample at both age 2 and age 5 in the NFBC1986. Height was measured on a subsample of the BCS1970 cohort at age 2 and the full sample at age 5. The main analyses used height at age 2 z-score for both the NFBC1986 and the CLHNS and used the average of height at ages 2 and 5 z-scores for the BCS1970. These summary statistics reflect that same specification.

In Table 3 the dependent variable is years of schooling defined by highest educational attainment and the independent variable of interest is the height-for-age z-score. In regards to the NFBC1986 cohort (top panel), a 1 standard deviation increase in HAZ between ages 2 and 5 was associated with an additional 0.12 years of schooling in unadjusted (baseline) model. Including socioeconomic confounders diminished the association marginally to 0.11. However, when biological confounders were included the association was reduced and a 1 standard deviation increase in HAZ related to an additional .06 years of school (the 95% confidence interval includes 0). The association was larger in the BCS1970 cohort (middle panel). In the baseline specification, a 1 standard deviation increase in HAZ was associated with .29 additional years of schooling, which was reduced to .18 additional years of schooling when socioeconomic confounders were included in the specification. Fully adjusted for biological confounders, the association was .10. Of the three cohorts, the largest association between physical development and educational attainment was observed in the CLHNS cohort (bottom panel). The baseline specification yielded an association of 1.027, while controlling for socioeconomic confounders reduced the association to .55. In the fully adjusted model, a 1 standard deviation increase in HAZ was associated with an additional .53 years of schooling.

**Table 3:** Physical Early Life Development and Educational Attainment.

Physical Early Child Development and Later-life Completed Years of Schooling for Each Cohort			
	*Dependent Variable: Years of schooling*		
	Baseline	Min. Adjustment	Full Adjustment
***1985–86 Northern Finland Birth Cohort (NFBC1986)***			
***Linear Model*:**			
Height for age z-score (ages 2–5)	0.124	0.114	0.057
	(0.028, 0.219)	(0.019, 0.209)	(-0.046, 0.160)
Number of observations	4,003	4,003	4,003
R-squared	0.045	0.053	0.061
***1970 British Cohort Study (BCS1970)***			
***Linear Model*:**			
Height for age z-score (ages 2–5)	0.287	0.176	0.103
	(0.224, 0.350)	(0.118, 0.234)	(0.042, 0.164)
Number of observations	6,533	6,533	6,533
R-squared	0.016	0.169	0.192
***1983–84 Cebu Longitudinal Health and Nutrition Survey (CLHNS)***			
***Linear Model*:**			
Height for age z-score (ages 2–5)	1.022	0.547	0.527
	(0.827, 1.218)	(0.352, 0.743)	(0.318, 0.737)
Number of observations	1,886	1,886	1,886
R-squared	0.061	0.168	0.177

Notes: Baseline regressions included gender fixed effects. Minimal adjustment regressions also included socio-economic information including mother's and father's years of education, and father's social class by occupation (1–6: non-manual skilled—unemployed). Full adjustments also included low birth weight indicator, mother's height, number of previous pregnancies, age at childbirth, and smoking behavior during pregnancy. Regional indicators were included for each regression (baseline, min. adj., full adj.) of the BCS1970 and NFBC1986. Statistics shown in parentheses are 95% confidence intervals.

Table 4 presents the associations between cognitive development and educational attainment for each cohort. In the NFBC1986 cohort data (top panel), the baseline specification demonstrated that a 1 standard deviation increase in cognitive development score was associated with an additional .26 years of schooling. Including socioeconomic confounders reduced the association to .23, and including biological confounders further reduced the association to .22. The association was substantially larger in the BCS1970 cohort (middle panel); a 1 standard deviation increase in cognitive development score was associated with an additional .87 years of schooling in the baseline specification, .63 in the minimally adjusted specification, and .58 in the fully adjusted specification. However, the largest association was observed in the CLHNS cohort (bottom panel). In the CLHNS, a 1 standard deviation increase in cognitive development score was associated with an additional 1.58 years of schooling in the baseline specification, 1.10 in the minimally adjusted specification, and 1.08 in the fully adjusted specification.

**Table 4:** Cognitive Early Life Development and Educational Attainment.

Cognitive Early Child Development and Later-life Completed Years of Schooling for Each Cohort			
	Dependent Variable: Years of schooling		
	Baseline	Min. Adjustment	Full Adjustment
***1985–86 Northern Finland Birth Cohort (NFBC1986)***			
***Linear Model*:**			
Std. cognitive score	0.261	0.233	0.218
	(0.168, 0.355)	(0.138, 0.327)	(0.124, 0.312)
Number of observations	4,003	4,003	4,003
R-squared	0.049	0.057	0.067
***1970 British Cohort Study (BCS1970)***			
***Linear Model*:**			
Std. cognitive score	0.870	0.634	0.583
	(0.805, 0.936)	(0.570, 0.697)	(0.519, 0.646)
Number of observations	6,533	6,533	6,533
R-squared	0.098	0.211	0.229
***1983–84 Cebu Longitudinal Health and Nutrition Survey (CLHNS)***			
***Linear Model*:**			
Std. cognitive score	1.576	1.102	1.078
	(1.389, 1.763)	(0.908, 1.297)	(0.883, 1.273)
Number of observations	1,886	1,886	1,886
R-squared	0.136	0.205	0.213

Notes: Baseline regressions included gender fixed effects. Minimal adjustment regressions also included socio-economic information including mother's and father's years of education, and father's social class by occupation (1–6: non-manual skilled—unemployed). Full adjustments also included low birth weight indicator, mother's height, number of previous pregnancies, age at childbirth, and smoking behavior during pregnancy. Regional indicators were included for each regression (baseline, min. adj., full adj.) of the BCS1970 and NFBC1986. Statistics shown in parentheses are 95% confidence intervals.

Table 5 displays the jointly estimated associations between physical and cognitive development and later life educational attainment for each cohort. Focusing on the fully adjusted specifications for each cohort, a 1 standard deviation increase in HAZ in the NFBC1986 cohort (top panel) was associated with .05 additional years of schooling and a 1 standard deviation increase in cognitive development score was associated with an additional .21 years of educational attainment. Neither of these jointly estimated associations were substantially different from separately estimated associations displayed in Tables 3 and 4 (HAZ: .06, cognitive development: .22). In the BCS1970 cohort (middle panel) only the HAZ-schooling association was marginally different when jointly estimated. Jointly estimated, a 1 standard deviation increase in HAZ was associated with an additional .08 years of schooling, in contrast to the separately estimated association of .11 (a 25% reduction). A 1 standard deviation increase in cognitive development score was associated with an additional .58 years of schooling, similar to the separately estimated association. The reduction in the HAZ-schooling association was similar (30%) in the CLHNS when jointly estimated. In the CLHNS (bottom panel), a 1 standard deviation increase in HAZ was associated with .37 additional years of schooling—reduced from .53 when separately estimated. However, as in the other cohorts, the separately estimated cognitive development-schooling association was similar to the separately estimated association in the CLHNS: a 1 standard deviation increase in cognitive development score was associated with an additional 1.02 years of schooling.

**Table 5:** Physical and Cognitive Early Life Development and Educational Attainment.

Physical and Cognitive Early Child Development and Later-life Completed Years of Schooling for Each Cohort			
	Dependent Variable: Years of schooling		
	Baseline	Min. Adjustment	Full Adjustment
***1985–86 Northern Finland Birth Cohort (NFBC1986)***			
***Linear Model*:**			
Height for age z-score (ages 2–5)	0.115	0.107	0.045
	(0.019, 0.210)	(0.011, 0.202)	(-0.059, 0.149)
Std. cognitive score	0.251	0.221	0.209
	(0.157, 0.346)	(0.126, 0.316)	(0.114, 0.304)
Number of observations	4003	4003	4003
R-squared	0.052	0.060	0.067
***1970 British Cohort Study (BCS1970)***			
***Linear Model*:**			
Height for age z-score (ages 2–5)	0.200	0.128	0.077
	(0.140, 0.260)	(0.071, 0.184)	(0.017, 0.136)
Std. cognitive score	0.846	0.621	0.579
	(0.780, 0.912)	(0.558, 0.685)	(0.515, 0.642)
Number of observations	6533	6533	6533
R-squared	0.103	0.213	0.230
***1983–84 Cebu Longitudinal Health and Nutrition Survey (CLHNS)***			
***Linear Model*:**			
Height for age z-score (ages 2–5)	0.626	0.372	0.367
	(0.432, 0.820)	(0.178, 0.566)	(0.160, 0.575)
Std. cognitive score	1.404	1.036	1.023
	(1.211, 1.596)	(0.839, 1.233)	(0.826, 1.220)
Number of observations	1886	1886	1886
R-squared	0.154	0.211	0.218

Notes: Baseline regressions included gender fixed effects. Minimal adjustment regressions also included socio-economic information including mother's and father's years of education, and father's social class by occupation (1–6: non-manual skilled—unemployed). Full adjustments also included low birth weight indicator, mother's height, number of previous pregnancies, age at childbirth, and smoking behavior during pregnancy. Regional indicators were included for each regression (baseline, min. adj., full adj.) of the BCS1970 and NFBC1986. Statistics shown in parentheses are 95% confidence intervals.

**Fig 2:**
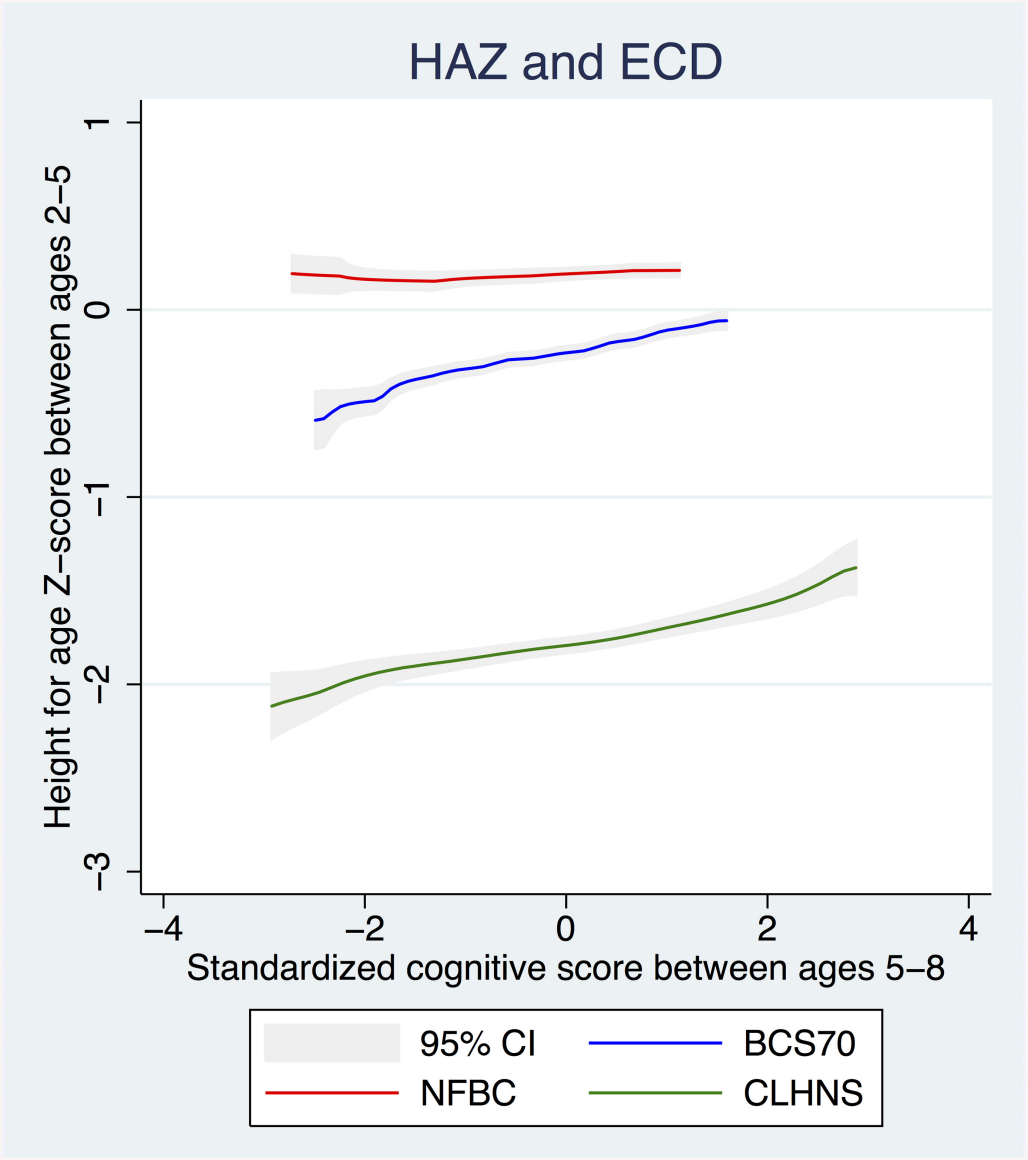
Height for age z-score and early life cognitive development. The shape of the relationship between height for age z-score and early life cognitive development in each of the 3 cohorts, including 95% confidence intervals.

**Fig 3:**
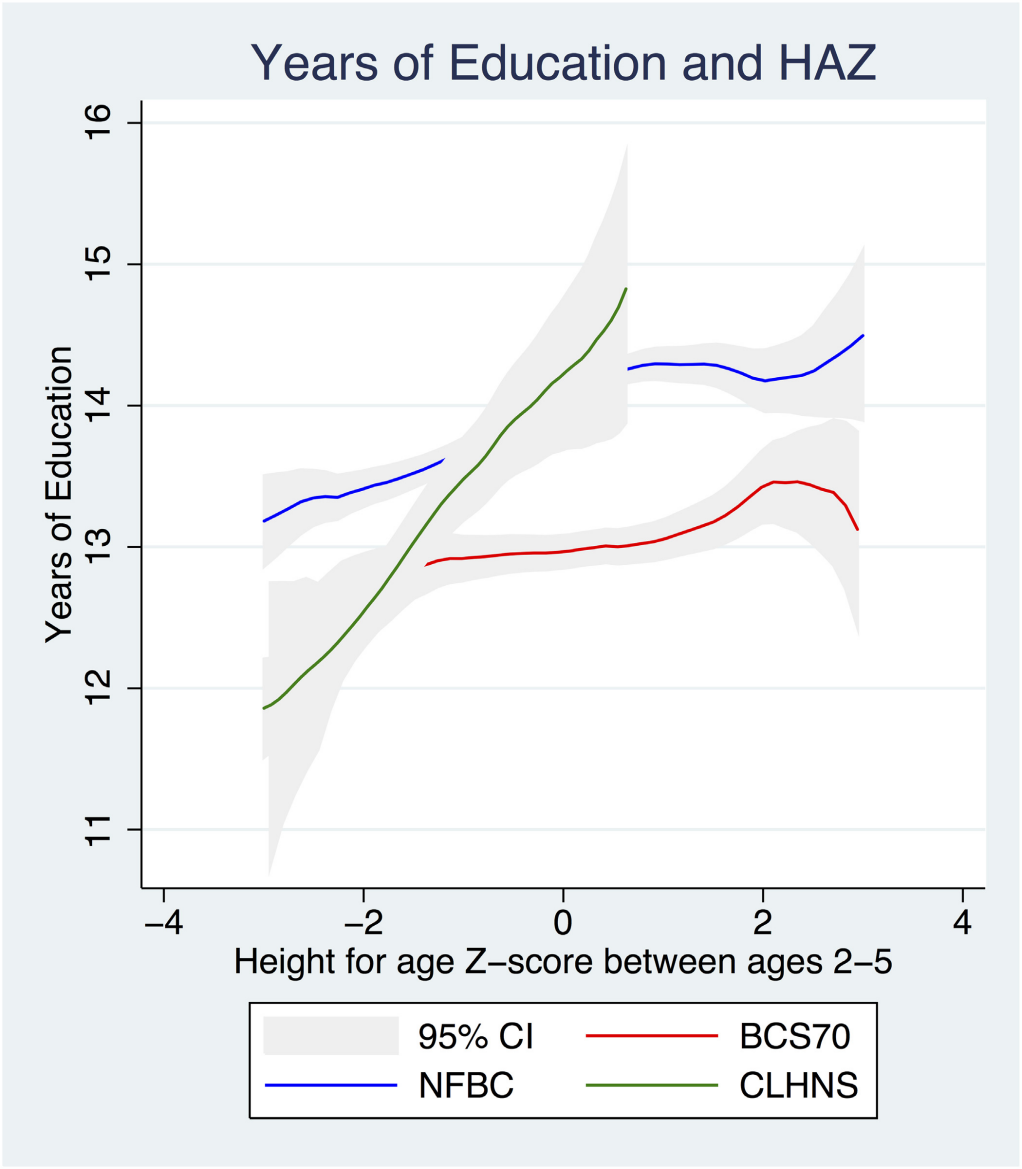
Height for age z-score and educational attainment. The shape of the relationship between height for age z-score and educational attainment in each of the 3 cohorts, including 95% confidence intervals.

**Fig 4:**
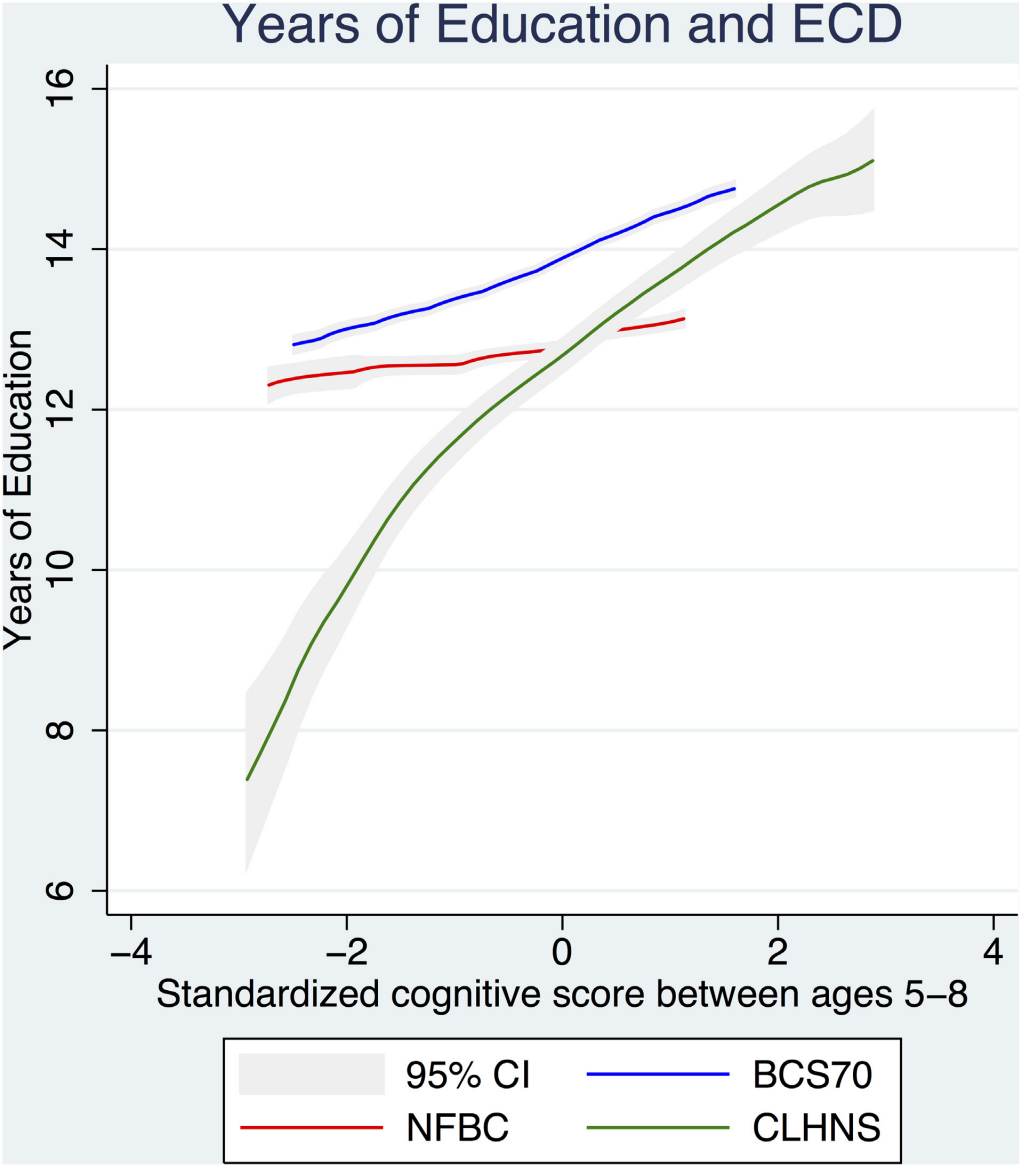
Early life cognitive development and educational attainment. The shape of the relationship between early life cognitive development and educational attainment in each of the 3 cohorts, including 95% confidence intervals.

The shapes of the associations between physical and cognitive development and years of schooling in each cohort are displayed in Figs 2–4. Fig 2 shows the relationship between HAZ and cognitive development in each cohort. The relationships were linear in each cohort with similar slopes in the CLHNS and BCS1970 and a slope near to zero in the NFBC1986. The significant difference between the three cohorts was in level of HAZ; the highest level of HAZ was observed in the NFBC1986, next in the BCS1970, and the lowest in the CLHNS. Fig 3 shows the relationship between HAZ and years of schooling in each cohort. Generally, the relationships in each cohort were linear across the HAZ distribution. The NFBC1986 demonstrated a potentially non-linear relationship with a greater slope at lower levels of the HAZ distribution; however estimation of the relationship by inclusion of higher order polynomials in the multivariate regression did not demonstrate a statistically significant non-linear relationship. Across cohorts, the most significant difference appeared between the slope of the CLHNS and the NFBC1986/BCS1970. The slope of the relationship was much larger in the CLHNS than in both the NFBC1986 and the BCS1970. Fig 4 shows the relationship between cognitive development and years of schooling in each cohort. While slightly logarithmic in the CLHNS, the relationship was generally linear. Again, the most significant difference appeared between the slope of the CLHNS and the NFBC1986/BCS1970. For values of cognitive development below zero (i.e., below the sample mean), the slope of the relationship was much larger in the CLHNS than in both the NFBC1986 and the BCS1970.

## Discussion

The analyses presented in this paper have yielded four main results. First, both physical and cognitive development predicted later life educational attainment in each cohort, with the strongest associations for both factors in the CLHNS. Second, in each cohort and across all specifications, the associations between cognition and schooling were stronger than the associations between schooling and physical development. Third, jointly estimating the physical development-schooling and cognitive development-schooling associations did not alter the cognitive development-schooling association but did diminish the physical development-schooling association in two of the three cohorts. Last, the strength of the associations was heterogeneous across contexts, with the strongest associations observed in the CLHNS and weakest in the NFBC1986.

Overall, the results indicated that physical and cognitive development each separately contributes to educational attainment. Given the generally high correlation between the two domains of ECD previous studies focusing only on the link between physical development and schooling have likely overstated the importance of physical development for educational attainment.^10-14^ Furthermore, because we found substantial heterogeneity between cohorts, caution is required in generalizing this relationship cross-contextually. While cognitive development consistently demonstrated larger associations with educational attainment in each cohort, the difference between the cognitive development-schooling association and the physical development-schooling association varied widely by context.

**Fig 5:**
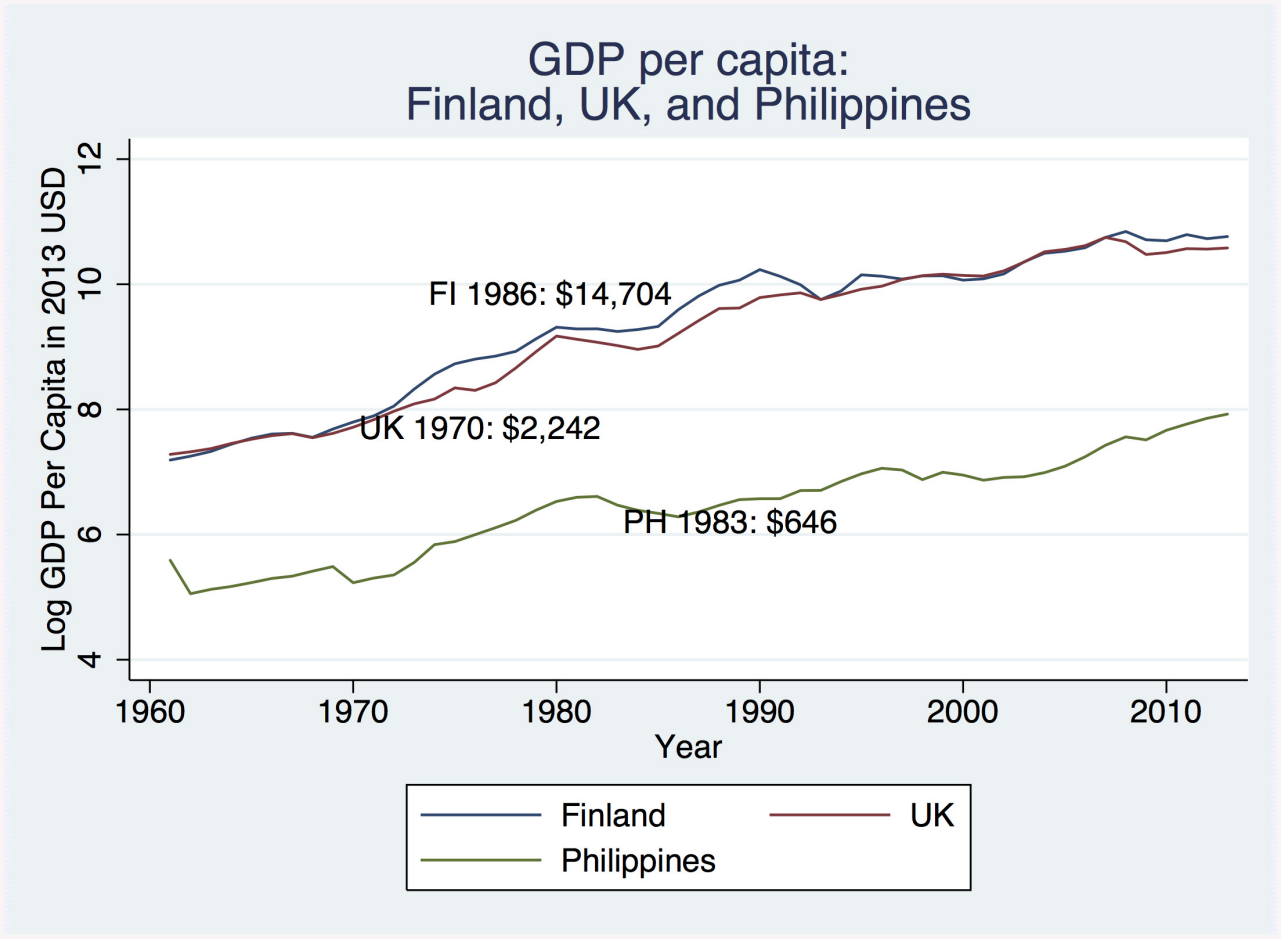
Log of GDP per capita of countries represented by each cohort study.

Economic differences were likely drivers of observed contextual heterogeneity. The three cohorts represented different levels of economic development as described by per capita gross domestic product (GDP). Fig 5 displays the log of per capita GDP for the countries of each cohort between 1960 and 2013 using data obtained from the World Bank World Development Indicators. While the time periods of each study differ, they identify three distinct levels of economic development. The Philippines in 1983–84 represented the lowest level of economic development with a per capita GDP of $646 (in 2013 USD), the United Kingdom in 1970 the middle level with a per capita GDP of $2,242 (250% more than the Philippines in 1983), and Finland in 1985–86 the highest level of development with a per capita GDP of $14,705 (550% more than the UK in 1970). The observed magnitudes of the physical development-schooling association mirrored the national economic development: the largest associations—and, consequently, returns to investment—are observed in the least economically developed context, and the smallest associations in the most developed.

While economic conditions seem related to the associations found across cohorts, both temporal and institutional influences are likely also key factors in explaining the observed differences. The heterogeneity across contexts indicates that the effects of physical growth and cognitive development on educational attainment are likely modified by economic conditions and educational systems. Confirming previous studies of health and nutritional investments in developing contexts, the patterns of associations across cohorts suggest greater returns on investments made to early physical development in the lowest income settings and that returns diminish as income and economic development increases [33,34]. It is also possible that improvements in physical growth are particularly important in settings like the Philippines, where a large fraction of children fall behind their expected age-specific trajectories.

Contextual patterns in the observed relationship between cognitive development and schooling are similarly associated with economic development and stem from nutritional, stimulatory, and other types of deprivation common in low income settings [35]. Consequently, benefits to investments such as early educational programs or home visits aimed at cognitive skill development may differ across contexts. While 1986 Finnish GDP per capita in 1986 exceeded 1970 British GDP per capita in by 550%, if, as has been postulated, both nations were sufficiently developed to surpass a developmental threshold where adequate nutrition and infection control exist [36], temporal and institutional differences may underlie observed differences at higher income levels. As seen in Fig 5, the per capita GDP of the UK and Finland are very similar in each year. While 1970 UK is less economically developed than 1986 Finland, 1970 UK is also less developed medically, scientifically, and in other ways that may impact the translation of early physical and cognitive development to educational attainment. Additionally, institutional differences factor in the differences between the BCS1970 and the NFBC1986. The UK educational system is merit based while the Finnish educational system downplays early development and provides a flexible learning environment for children to succeed at varying rates [37]. Perhaps as a result we observe less divergence in the schooling outcomes of children with high and low early cognitive development in the NFBC1986 than we do in the BCS1970.

Despite the strengths of this study, the conclusions both within and across cohorts are limited for a variety of reasons. First, in order to estimate identical models for each cohort, the set of confounders is limited and may result in confounding from unobserved or excluded information on parental investments, environmental conditions and other characteristics. To examine this possibility we performed additional estimations which included confounders such as parent’s marital status, number of antenatal visits to healthcare provider, gestational length, mother’s employment status, and delivery complications which are available in the BCS1970 and CLHNS. The inclusion of each of these confounders did not significantly alter the associations observed in the BCS1970 and CLHNS cohorts suggesting that the fully adjusted model may have adequately captured at least some unobserved heterogeneity associated with schooling.

The second limit to the study’s conclusions is that height and cognition are not measured at the same point in time for each of the cohorts. Height at age 2 is measured for the full NFBC1986 and CLHNS cohorts and a subsample of the BCS1970 cohort, while the full BCS1970 sample is observed at age 5. The same analysis has also been performed using HAZ at age 5, and average HAZ between ages 2 and 5 and the results do not substantially differ. Cognition is measured at age 5 in the BCS1970, and between ages 7 and 8 in both the NFBC1986 and CLHNS. It is possible that early preschool or school exposure may have affected these scores in the NFBC1986 and CLHNS cohorts. However, it is not clear whether early schooling increases or decreases the cognitive gap between children; if schooling allows less developed children to catch up, the later measures would underestimate early differentials; if schooling increases the gap by focusing on the most talented students, the opposite may be true.

Another limitation of the study is that the measures of cognition likely contain error and may not be completely comparable. Where available, alternative specifications of cognitive development have been assessed and demonstrate strong similarity to the results presented. However, error is particularly salient in the NFBC1986 measure of cognition derived from parental reports of their child’s understanding of spatial and temporal concepts. And despite our efforts to utilize the most comparable measures, cognition is measured differently in each cohort, making the levels incomparable across cohorts and prevented us from pooling the three cohorts in order to estimate an overall association between cognitive development and schooling. Fourth, the number and representativeness of the cohorts are limited. We are unaware of additional prospective cohorts containing the requisite measures; we generated only three points along the potentially non-linear ECD-schooling relationships implying that the results should not be generalized more broadly.

An additional limitation to the study’s conclusions is that while the baseline samples are representative of most of the UK, the two northernmost Finnish provinces, and one Philippine metropolitan area, each cohort contains a significant amount of attrition which may be related to early physical and cognitive development. While unassociated with observed baseline characteristics in the NFBC1986 [37], attrition in the BCS1970 was selective on socioeconomic status (specifically father’s social class by occupation) [38] and attrition in the CLHNS was selective on socioeconomic status (both parental education and father’s social class by occupation) [39]. However, these differences are relatively minor; for instance, the proportion of mothers with primary or less education in the original (full) CLHNS sample differs by less than 4 percentage points from the proportion observed in the sample of children followed up; for mother’s with secondary education the difference is 1 percentage point. While attrition does not appear to play a key role in biasing the sample based on observed characteristics, the degree to which the baseline and analytic samples differed on unobserved characteristics remains unknown.

In spite of these limitations, the results of this study provide insight into the relationships between physical development, cognitive development, and educational attainment across multiple contexts spanning different institutions and levels of economic development. Overall, the results indicate that both physical and cognitive development are separately and jointly important for children’s subsequent educational attainment, with cognitive development playing a particularly strong role. Previous studies likely overstate the importance of physical development due to correlation with cognitive development, which is consistently a stronger determinant of educational attainment. The heterogeneity in the associations across contexts reflects both economic and institutional conditions.
